# MET kinase inhibitor reverses resistance to entrectinib induced by hepatocyte growth factor in tumors with NTRK1 or ROS1 rearrangements

**DOI:** 10.1002/cam4.5342

**Published:** 2022-11-23

**Authors:** Yohei Takumi, Sachiko Arai, Chiaki Suzuki, Koji Fukuda, Akihiro Nishiyama, Shinji Takeuchi, Hiroki Sato, Kunio Matsumoto, Kenji Sugio, Seiji Yano

**Affiliations:** ^1^ Division of Medical Oncology Cancer Research Institute, Kanazawa University Kanazawa Japan; ^2^ Department of Thoracic and Breast Surgery Faculty of Medicine Oita University Yufu Japan; ^3^ Division of Tumor Dynamics and Regulation Cancer Research Institute, Kanazawa University Kanazawa Japan; ^4^ Department of Respiratory Medicine Faculty of Medicine Institute of Medical, Pharmaceutical, and Health Sciences Kanazawa Japan; ^5^ WPI‐Nano Life Science Institute (WPI‐Nano LSI) Kanazawa University Kanazawa Japan

**Keywords:** entrectinib, HGF, MET, NTRK, ROS1

## Abstract

**Background:**

Entrectinib is an effective drug for treating solid tumors with NTRK gene rearrangement and non‐small cell lung cancer (NSCLC) with ROS1 gene rearrangement. However, its efficacy is limited by tolerance and acquired resistance, the mechanisms of which are not fully understood. The growth factors produced by the tumor microenvironment, including hepatocyte growth factor (HGF) produced by tumor‐associated fibroblasts, critically affect the sensitivity to targeted drugs.

**Methods:**

We investigated whether growth factors that can be produced by the microenvironment affect sensitivity of NTRK1‐rearranged colon cancer KM12SM cells and ROS1‐rearranged NSCLC HCC78 cells to entrectinib both in vitro and in vivo.

**Results:**

Among the growth factors assessed, HGF most potently induced entrectinib resistance in KM12SM and HCC78 cells by activating its receptor MET. HGF‐induced entrectinib resistance was reversed by the active‐HGF‐specific macrocyclic peptide HiP‐8 and the MET kinase inhibitor capmatinib in vitro. In addition, HGF‐producing fibroblasts promoted entrectinib resistance in vitro (culture model) and in vivo (subcutaneous tumor model). The use of capmatinib circumvented entrectinib resistance in a subcutaneous tumor model inoculated with KM12SM and HGF‐producing fibroblasts.

**Conclusion:**

Our findings suggest that growth factors in the tumor microenvironment, such as HGF, may induce resistance to entrectinib in tumors with NTRK1 or ROS1 rearrangements. Our results further suggest that optimally co‐administering inhibitors of resistance‐inducing growth factors may maximize the therapeutic efficacy of entrectinib.

## 1 INTRODUCTION

The neurotrophic tropomyosin receptor kinases (NTRKs) 1/2/3 encode tropomyosin receptor kinases (TRKs) A/B/C, respectively. Wild‐type TRKs bind to their ligands (neurotrophic factors such as nerve growth factor, brain‐derived neurotrophic factor, and neurotrophins) to transmit intracellular signals and regulate neuronal differentiation and survival in the central and peripheral nervous systems.^1^ The ROS proto‐oncogene 1 (*ROS1*), located on chromosome 6q22, also encodes a receptor tyrosine kinase. The function of wild‐type ROS1 in human cellular physiology remains unclear, and its ligand is undefined.^2^

Recently, gene rearrangements of tyrosine kinase receptors, including NTRKs and ROS1, have been identified as druggable oncogene drivers in various types of solid tumors. TRK and ROS1 fusion proteins constitutively activate tyrosine kinases and continue to emit survival and proliferation signals, resulting in overgrowth and prolonged survival of tumor cells.^3^, ^4^, ^5^ NTRK rearrangements have been identified in a variety of cancers, with positivity rates varying depending on the type of cancer. ROS1 rearrangement has been reported in 1–2% of non‐small cell lung cancers (NSCLC).^6^

Entrectinib is a molecularly targeted drug that selectively inhibits TRK‐A/B/C and ROS1. It diminishes cell proliferation by inhibiting phosphorylation of TRKs or ROS1 fusion proteins and signaling molecules located downstream of TRK or ROS1 signaling.^7^ The drug has been shown to be effective and feasible in treating patients with NTRK‐rearranged solid tumors and advanced ROS1 rearranged NSCLC, in an integrated analysis of relevant clinical trials.^8^, ^9^

However, as with other molecularly targeted drugs, tumors with NTRK or ROS1 rearrangements also develop resistance to entrectinib, even after an initial response. Kinase domain mutations in NTRKs and ROS1, including NTRK1‐G595R, NTRK3‐G623R, and ROS1‐G2032R, have been reported to be involved in on‐target resistance mechanisms. In contrast, MET amplification, KRAS mutation, BRAF‐V600E mutation, and IGF‐1R activation have been reported as off‐target resistance mechanisms.^10^, ^11^, ^12^

In addition, crosstalk between tumor cells and the surrounding tumor environment can affect tumor progression and sensitivity to drug.^13^ We previously reported that hepatocyte growth factor (HGF), predominantly produced by fibroblasts, plays an important role in lung cancer cell lines that are resistant to EGFR‐TKIs.^14^ We also demonstrated that MET inhibitors can overcome HGF‐induced resistance to EGFR tyrosine kinase inhibitors (TKIs) in EGFR‐mutated lung cancer cell lines.^15^

Here, we investigated whether growth factors produced in the tumor microenvironment can induce resistance to entrectinib in tumor cells with NTRK1 or ROS1 rearrangements. We found that HGF (produced by fibroblasts) induced entrectinib resistance in tumor cells with NTRK1 or ROS1 rearrangement and that HGF‐induced resistance was reversed by combination with HGF/MET inhibitors.

## 2 MATERIAL AND METHODS

### 2.1 Cell culture and reagents

The NTRK fusion human colorectal cancer cell line KM12SM was kindly gifted by Dr. I. J. Fidler (MD Anderson Cancer Center) to our laboratory in 1999. KM12SM cells, a highly metastatic variant of KM12C cells, have the TPM3‐NTRK1 gene rearrangement. HCC78 cells were obtained from American Type Culture Collection. The PC‐9 cell line with an *EGFR* exon 19 deletion was obtained from the RIKEN Cell Bank. A925L cells with EML4‐ALK variant 5a (E2:A20) were kindly provided by Dr. H. Uramoto (Kanazawa Medical University). Human lung fibroblasts MRC‐5 and IMR‐90 were obtained from RIKEN Cell Bank. The KM12SM and HCC78 cell lines were cultured in RPMI 1640 with 10% fetal bovine serum (FBS), penicillin (100 U/ml), and streptomycin (10 mg/ml) in a humidified, 5% CO2 incubator at 37°C. The IMR‐90 and MRC‐5 cell lines were maintained in DMEM containing 10% FBS, penicillin (100 U/ml), and streptomycin (10 mg/ml). Entrectinib and capmatinib were obtained from Selleck Chemicals (Houston, TX, USA). Recombinant NRG, HGF, IGF‐1, FGF2, and amphiregulin were purchased from R&D Systems (Minneapolis, MN, USA). Goat anti‐human HGF neutralizing antibody and control goat IgG were obtained from R&D Systems as well.

### 2.2 Cell viability assay

MTT assay was used to measure cell viability. Tumor cells were spread on 96‐well plates at a density of 2 × 10^3^ cells/100 μl RPMI‐ 1640, containing 10% FBS and were cultured overnight t. The next day, the indicated compound was added to each well and incubated for 72 h. MTT solution (2 mg/ml; Sigma) was used to measure cell growth, as previously described. Percentage growth was determined by comparison with untreated controls. Experiments were independently repeated at least three times in triplicate.

### 2.3 Antibodies and Western blotting

Cell extracts (15 μg) were separated by SDS‐PAGE (Bio‐Rad), and separated proteins were transferred to Immun‐Blot PVDF membranes (Bio‐Rad). The membranes were incubated with primary antibodies overnight at 4°C. The primary antibodies used in this study were as follows: TRKA (14G6), phosphor‐TRKA (Tyr490), MET (25H2), MET (D1C2), phosphor‐MET (Tyr1234/1235), Gab1, phosphor‐Gab1 (Tyr627), EGFR (D38B1), FGFR1 (D8E4), IGF‐1 Receptor β (D23H3), β‐actin (1:1000; Cell Signaling Technology). After treating the membrane with primary antibody, it was cultured with species‐specific HRP‐conjugated secondary antibody for 1 h at room temperature. SuperSignal West Dura Extended Duration Substrate (Pierce Biotechnology) was used to detect immunoreactive bands.

### 2.4 Cytokine production

Cells (2 × 10^5^) were incubated in RPMI‐1640 or DMEM medium with 10% FBS for 24 h, washed with PBS, and cultured in 2 ml of the same medium for 48 h. The supernatant was collected and centrifuged. The levels of HGF and FGF2 were measured using a Quantikine ELISA kit and NRG was quantified by Human NRG1‐beta 1/HRG1‐beta 1 DuoSet ELISA (R&D Systems) for targeted cytokines in accordance with the manufacturer's protocols. All experiments were performed in triplicate. Color intensity was measured at 450 nm using a spectrophotometric plate reader. Cytokine concentrations were measured by comparing them to the standard curve.

### 2.5 Co‐culture of tumor cells with fibroblasts

Transwell chambers filters with 8 μm pores were used for cells co‐culture. Fibroblasts (5 × 10^4^ cells/300 μl), in the presence or absence of 1 h pre‐treatment with control IgG or anti‐HGF neutralizing antibody (2 μg/ml; R&D Systems), were spread on the upper chamber, and cancer cells (8 × 10^3^ cells/700 μl) in the presence or absence of entrectinib (10 nmol/L) were spread on the lower chamber. After 72 h, the upper chamber was removed and MTT assay was used to measure cell proliferation of lower chamber. Percentage growth was determined by comparison with untreated controls. Experiments were independently performed at least three times in triplicate.

### 2.6 Tumor cell inoculation in mice with SCID

Five‐week‐old male SCID mice were obtained from Clea Japan. SCID mice were injected subcutaneously with KM12SM cells (5 × 10^6^) mixed in the presence or absence of MRC‐5 cells (5 × 10^6^) in their back. Four days later, mice were divided into four groups and drugs (entrectinib [25 mg/kg/d] and capmatinib [15 mg/kg/d]) were administered by oral administration. The tumor volume was calculated as mm^3^ = width^2^ × length/2 Tumor measurements and weight measurements were taken once every couple of days. The study protocol was performed in compliance with the Guidelines for the Institute for Experimental Animals, Kanazawa University Advanced Science Research Center.

### 2.7 HiP‐8 inhibits drug‐resistance activity induce by HGF using EZ‐BindShut

HiP‐8 was synthesized as described previously.^16^ For the entrectinib resistance assay, KM12SM and HCC78 cells were spread on each well of a 24‐well suspension culture plate (EZ‐BindShut II, IWAKI) in RPMI medium including 10% FBS. After 24 h of incubation, the cells were treated with HGF (30 ng/ml) and HiP‐8 (1 μM) with or without entrectinib (KM12SM: 10 nM, HCC78: 1 μM), and cultured for another 72 h. The hemocytometer was used to count surviving cells.

### 2.8 Statistical analysis

Differences between groups were analyzed using one‐way analysis of variance (ANOVA). All statistical analyses were performed using the GraphPad Prism ver. 9.1 (GraphPad Software, Inc.). Statistical significance was set at *p* < 0.05.

## 3 RESULTS

### 3.1 Various growth factors induced entrectinib resistance at different levels in tumor cells with NTRK‐ or ROS1‐rearrangement

**FIGURE 1:**
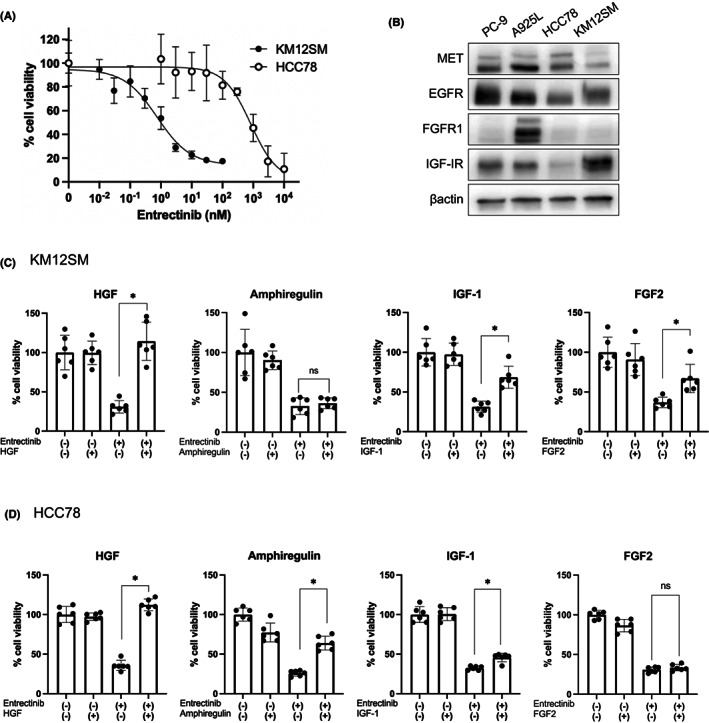
Various growth factors induced entrectinib resistance in tumor cells with NTRK1‐ or ROS1 rearrangement. (A) The effect of entrectinib on KM12SM and HCC78 cell lines. (B) The expression of major receptors determined by western bot in tumor cells. KM12SM (C) and HCC78 (D) cells were treated with 100 ng/ml of indicated receptor ligands for 72 h. Cell viability was measured using MTT assay thereafter.

KM12SM cells with TPM3‐NTRK1 and HCC78 cells with SLC34A‐ROS1 were sensitive to entrectinib, while IC_50_ value was much lower in KM12SM cells compared with HCC78 cells (0.75 nM vs. 828 nM) (Figure 1A). Compared with control human lung cancer cell lines PC‐9 and A925L, KM12SM and HCC78 cells expressed lower levels of EGFR and no detectable levels of FGFR1. HCC78 cells expressed a higher level of MET and KM12SM cells expressed a higher level of IGF‐1R (Figure 1B). We chose HGF, amphiregulin, IGF‐1, and FGF‐2 as growth factors that can be produced by microenvironments and examined their effects on susceptibility to entrectinib. In the microenvironment, it was reported that the major sources of HGF, amphiregulin, IGF‐1, and FGF‐2 are fibroblasts, leukocyte populations, cancer‐associated fibroblasts, and epithelial/endothelial cells, respectively.^17^, ^18^, ^19^ Tumor cells were treated with these growth factors at the concentration of 100 ng/ml. These growth factors did not remarkably affect the baseline viability of KM12SM or HCC78 cells. In KM12SM cells, HGF completely induced resistance (Figure 1C). While IGF‐1 and FGF‐2 partially induced resistance, amphiregulin did not induce resistance. In HCC78 cells, HGF completely induced resistance to entrectinib (Figure 1D). While amphiregulin and IGF‐1 partially induced resistance, FGF‐2 did not induce resistance.

These results indicate that although various growth factors could induce resistance at different levels depending on the tumor cell lines, HGF was the most potent in inducing entrectinib resistance in KM12SM and HCC78 cells under our experimental conditions.

### 3.2 HGF induced entrectinib resistance in tumor cells with NTRK1‐ or ROS1‐rearrangement

**FIGURE 2:**
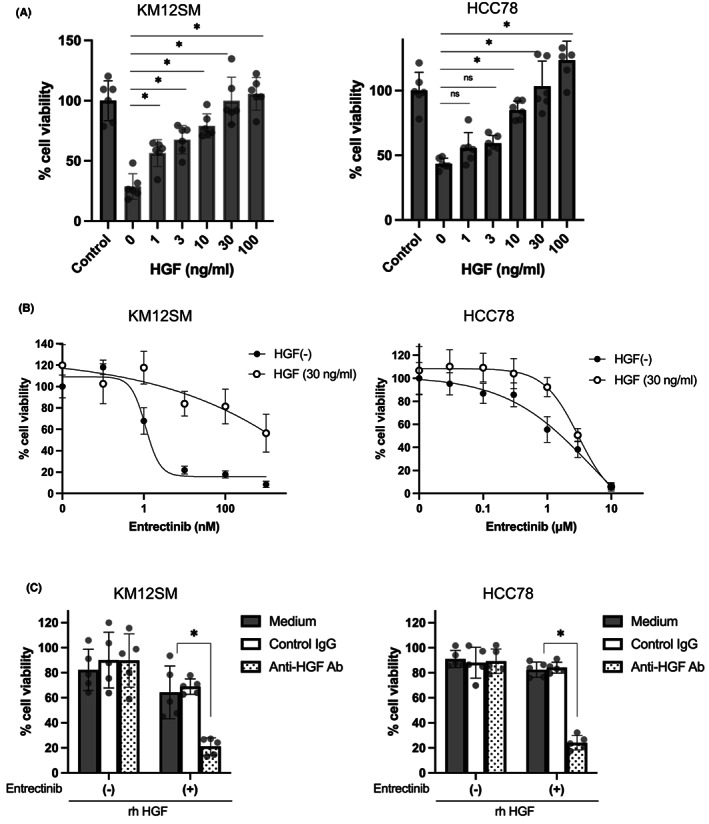
HGF induced entrectinib resistance in tumor cells with NTRK1‐ or ROS1‐rearrangement. (A) Different concentrations of HGF were added to the KM12SM and HCC78 cells with or without entrectinib, and MTT assay was used measure cell viability after 72 h. (B) KM12SM and HCC78 cells were cultured with the different concentrations of entrectinib in the presence or absence of HGF (30 ng/ml) for 72 h. (C) HGF (30 ng/ml) and control IgG or anti‐HGF antibody (2 μg/ml) were mixed for 1 h beforehand. The resultant solutions were added to cultures of KM12SM and HCC78 cells with or without entrectinib (10 nM for KM12SM, 1 μM for HCC78).

Next, we examined the effect of different concentrations of HGF on the induction of entrectinib resistance. HGF induced the resistance of KM12SM and HCC78 cells to entrectinib (10 nM for KM12SM and 1000 nM for HCC78) in a dose‐dependent manner, while higher concentrations of HGF were required for HCC78 cells to cause statistically significant resistance, compared with KM12SM cells (Figure 2A). HGF at 1 ng/ml and 10 ng/ml concentration discernibly increased the viability of KM12SM and HCC78 cells, respectively. On the other hand, HGF at 30 ng/ml induced resistance completely in both KM12SM and HCC78 cells (Figure 2B).

The effects of HGF (30 ng/ml) were reversed by an anti‐HGF neutralizing antibody (2 μg/ml), but not by control IgG (2 μg/ml) (Figure 2C). These results confirmed that HGF has the potential to induce entrectinib resistance in NTRK1‐ or ROS1 rearranged tumor cells.

### 3.3 HiP‐8 reversed entrectinib resistance induced by HGF

**FIGURE 3:**
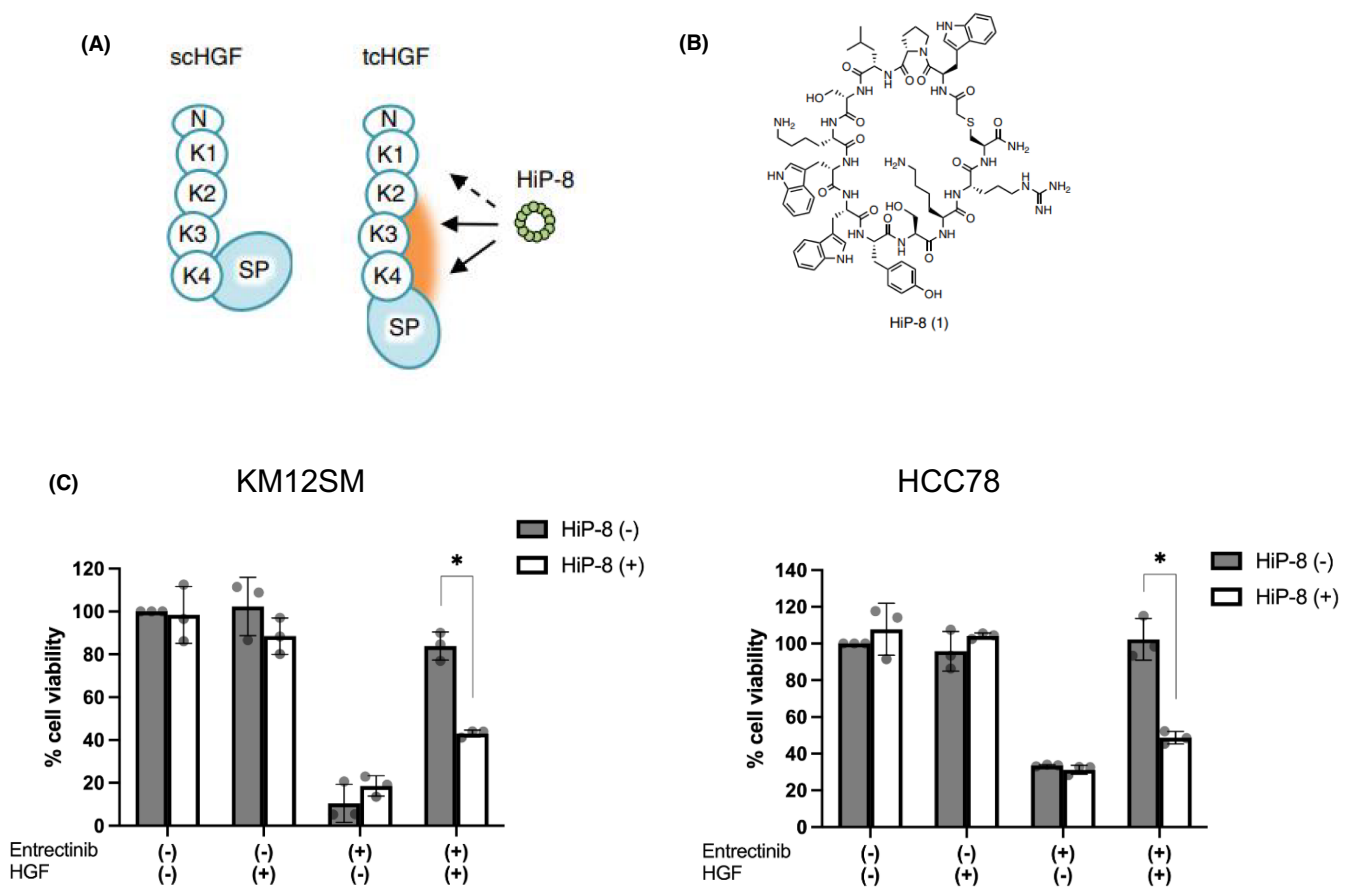
HiP‐8 reversed entrectinib resistance induced by HGF. (A) Scheme of interaction between HiP‐8 and HGF. (B) Structure of HiP‐8. (C) KM12SM and HCC78 cells were cultured with or without entrectinib (10 nM for KM12SM, 1 μM for HCC78), HGF (30 ng/ml) and HiP‐8 (1 μM) for 72 h. Bars indicate standard error.

HGF is composed of six individual domains: the amino terminus (N terminus; N), first to fourth kringle (K1‐K4), and carboxy terminus (C‐terminus) serine protease‐like (SP) domain. HGF is an inactive single‐chain polypeptide (scHGF) when secreted, but in the tumor microenvironment, scHGF is transformed into active two‐chain HGF (tcHGF) by specific serine protease‐mediated processing between the K4 domain and the SP domain (Figure 3A).^20^ HGF‐inhibitory peptide‐8 (HiP‐8) is an inhibitory macrocyclic peptide that specifically binds to tcHGF (Figure 3B). We have previously reported that HiP‐8 potently inhibits the HGF‐MET interaction.^16^ Therefore, we examined whether HiP‐8 reverses the effect of HGF on the sensitivity of KM12SM and HCC78 cells in 3D culture using EZ‐BindShut coated with a minimally adherent polymer. In this 3D culture model, HGF‐induced resistance to entrectinib, and HiP‐8 discernibly reversed the resistance in both KM12SM and HCC78 cells (Figure 3C). These results indicate the potential of HiP‐8 to circumvent the resistance induced by HGF.

### 3.4 Co‐culture with fibroblasts‐induced entrectinib resistance via HGF production

**FIGURE 4:**
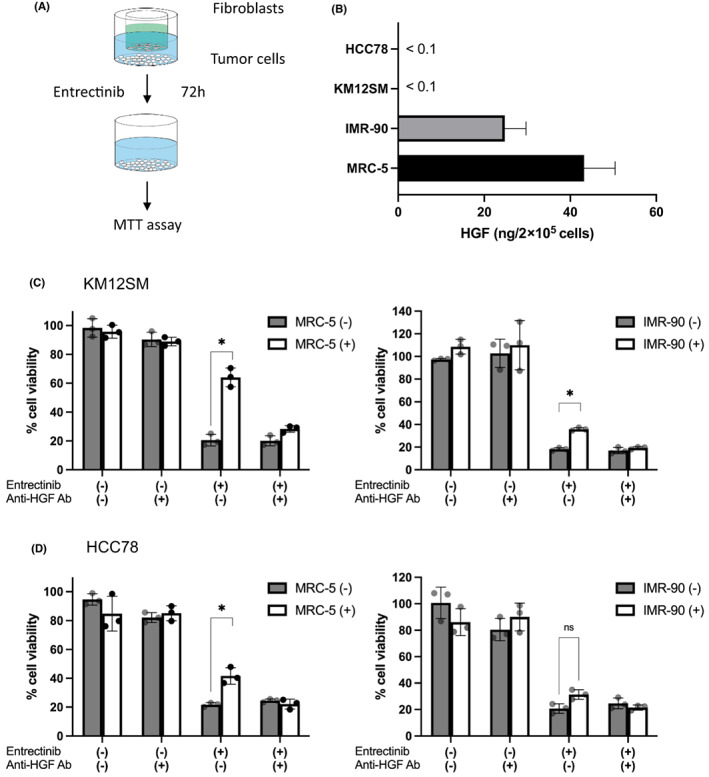
Co‐culture with fibroblasts induced entrectinib resistance via HGF production. (A) Transwell chambers filters with 8 μm pores were used. Fibroblasts (5 × 10^4^ cells/300 μl) were spread on the upper chamber, and tumor cells (8 × 10^3^ cells/700 μl) were spread on the lower chamber. (B) HGF production in tumor cells and fibroblast; After the cells were incubated in the culture medium for 48 h, the supernatants of the culture medium were collected. Levels of ligands in the collected supernatants were determined by ELISA. Error bars indicate SD of triplicate cultures. KM12SM (C) and HCC78 (D) cells were co‐cultured with MRC‐5 or IMR‐90 cells and anti‐HGF neutralizing antibody (2 μg/ml) in the presence or absence of entrectinib (10 ng/ml) for 72 h, and MTT solution was used to measure cell growth. Each sample was tested in triplicate, and each experiment was independently repeated at least three times.

We have previously reported that HGF induces resistance to erlotinib and gefitinib in EGFR‐mutated NSCLC cell lines HCC827and PC‐9 and that stromal fibroblasts are the major source of HGF production.^14^ Therefore, we investigated whether the susceptibility of KM12SM and HCC78 cells to entrectinib could be affected by fibroblasts, such as MRC‐5 and IMR‐90, using Transwell systems (Figure 4A). Both MRC‐5 and IMR‐90 cells, but not KM12SM or HCC78 cells, produced HGF in the culture supernatant, whereas MRC‐5 cells produced higher levels of HGF than IMR‐90 cells (Figure 4B). No detectable levels of amphiregulin, IGF‐1, or FGF‐2 were detected in the culture supernatants of KM12SM or HCC78 cells (data not shown). In the presence of fibroblasts, KM12SM cells became resistant to entrectinib (Figure 4C), while MRC‐5 cells were more potent than IMR‐90 cells. In contrast, HCC78 cells became resistant to entrectinib in the presence of MRC‐5 cells (Figure 4D). Co‐culture with IMR‐90 cells did not significantly induce entrectinib resistance, probably because of the lower HGF‐producing potential of IMR‐90 cells and less susceptibility of HCC78 cells to HGF (Figure 2B). The resistance caused by co‐culture with MRC‐5 or IMR‐90 cells was reversed by an anti‐HGF neutralizing antibody. These results suggest that fibroblasts may induce entrectinib resistance in KM12SM and HCC78 cells, predominantly by producing HGF.

### 3.5 MET kinase inhibitor reversed entrectinib resistance induced by HGF

**FIGURE 5:**
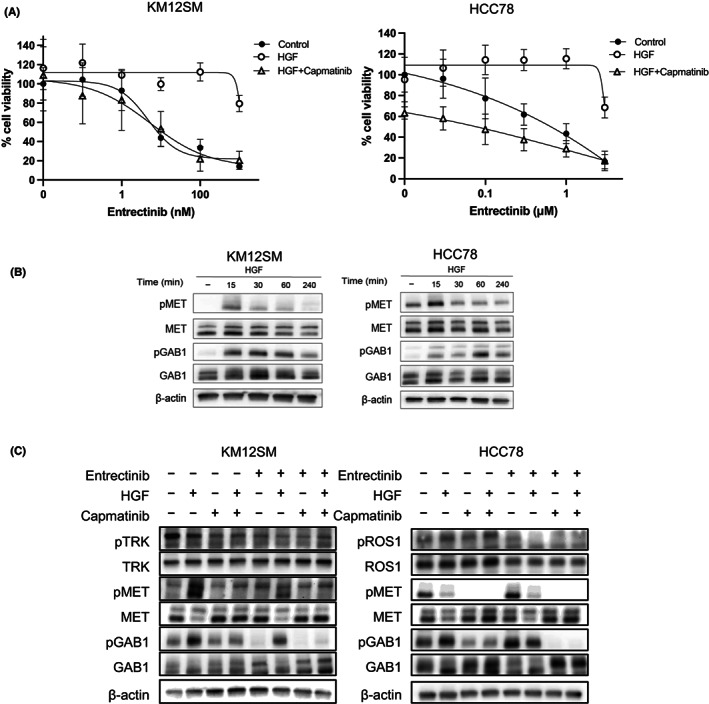
MET inhibitor reverses resistance to entrectinib induced by HGF. (A) KM12SM and HCC78 cells were cultured with different concentrations of entrectinib in the presence or absence of MET inhibitor capmatinib (100 nM) and HGF (30 ng/ml). (B) KM12SM and HCC78 cells were cultured with HGF (30 ng/ml) for 15, 30, 60, and 240 min. Immunoblotting was used for the protein detection. (C) KM12SM and HCC78 cells were treated in the presence or absence of entrectinib (10 nM for KM12SM, 1 μM for HCC78) and/or HGF (30 ng/ml) or capmatinib (100 nM) for 4 h. Immunoblotting was used for the protein detection.

We examined whether MET kinase inhibitors reversed the entrectinib resistance induced by HGF. We used capmatinib, an FDA‐approved drug, as a MET kinase inhibitor.^21^ Capmatinib alone did not suppress the viability of KM12SM cells, but inhibited the viability of HCC78 cells by 40% (Figure 5A). The combination of capmatinib and entrectinib reversed HGF‐induced resistance in KM12SM cells. Similarly, the combined use of capmatinib sensitized HCC78 cells to entrectinib in the presence of HGF.

Next, we explored the effect of HGF and capmatinib on signal transduction. HGF induced a remarkable increase in MET phosphorylation in 15 min in KM12SM and HCC78 cells (Figure 5B). MET phosphorylation induced by HGF decreased after 30 min in both cell lines. Interestingly, phosphorylation of GAB1, an adaptor protein for MET, was also induced in 15 min, and remained longer than 60 min. Under these experimental conditions, we investigated the effects of capmatinib in the presence or absence of entrectinib for 4 h.

In KM12SM cells, entrectinib inhibited TRK phosphorylation (Figure 5C). HGF increased MET phosphorylation as well as phosphorylation of GAB, suggesting that HGF restored survival signals via MET/GAB1. A similar trend was observed in HCC78 cells, but increased MET phosphorylation by HGF was not clearly observed. Since HGF activated MET/GAB1 pathway in entrectinib‐treated HCC78 cells, the timing of tumor cell lysate harvesting might not be optimal for detecting HGF‐induced MET phosphorylation. Collectively, these results indicate that capmatinib circumvented HGF‐induced entrectinib resistance by suppressing MET phosphorylation in KM12SM and HCC78 cells in vitro.

### 3.6 Use of MET inhibitor reversed fibroblast‐induced entrectinib resistance in vivo

**FIGURE 6:**
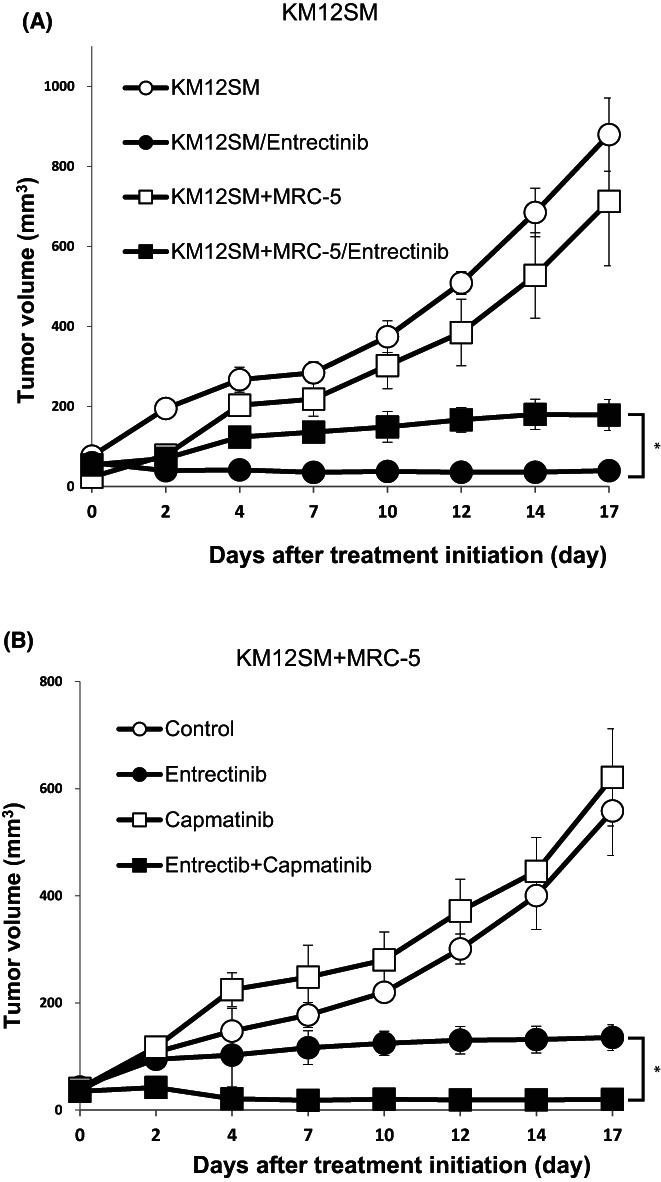
Combined used of MET inhibitor overcame fibroblast‐induced entrectinib resistance in vivo. (A) SCID mice were injected subcutaneously with KM12SM cells (5 × 10^6^) mixed with or without MRC‐5 cells (5 × 10^6^) in their back. Four days later, mice were divided into four groups and entrectinib (25 mg/kg/day) were given by oral administration. Bars indicate standard error. *p < 0.05 by Mann–Whitney test. (B) CID mice were injected subcutaneously with KM12SM cells (5 × 10^6^) mixed with or without MRC‐5 cells (5 × 10^6^) in their back. Mice were divided into four groups and received oral drugs (entrectinib (25 mg/kg/day), capmatinib (15 mg/kg/day)), starting on day 4. Bars indicate standard error. *p < 0.05 by Mann–Whitney test.

We investigated whether fibroblasts induced entrectinib resistance and whether the MET inhibitor circumvented the induced resistance in vivo. In the first set of in vivo experiments, KM12SM cells with or without fibroblast MRC‐5 were subcutaneously inoculated into SCID mice. The growth of KM12SM cells were not affected by co‐injection of MRC‐5 cells (Figure 6A). Mice injected subcutaneously with KM12SM alone showed prominent tumor regression after entrectinib treatment. The same treatment prevented tumor enlargement in mice injected with KM12SM and MRC‐5 cells, but did not cause tumor regression, indicating resistance of the tumors to entrectinib treatment in vivo.

In the second set of experiments, we inoculated a mixture of KM12SM and MRC‐5 cells into SCID mice (Figure 6B). Treatment with entrectinib alone inhibited tumor growth but not regression tumor size, consistent with the results obtained in the first set of experiments. While treatment with capmatinib alone did not affect the growth of tumors, The combination of entrectinib and capmatinib treatment caused tumor shrinkage. These results clearly indicate that the combined use of capmatinib could circumvent entrectinib resistance induced by HGF‐producing fibroblasts in vivo.

## 4 DISCUSSION

In this study, we showed that HGF, which can be produced by fibroblasts in the tumor microenvironment, induces entrectinib resistance by activating its receptor MET and downstream signaling pathways in NTRK1‐rearranged or ROS1‐rearranged tumor cells. The resistance was circumvented by the combined use of HGF/MET inhibitors, such as active‐HGF specific macrocyclic peptide HiP‐8 and MET kinase inhibitor capmatinib. Activation of bypass signaling mediated by the HGF‐MET pathway has been well recognized as a mechanism of resistance to targeted drugs in various cancers. In lung cancer, we and other groups reported that activation of HGF/MET causes resistance to EGFR‐TKIs and ALK‐TKIs in EGFR‐mutated NSCLC and ALK‐rearranged NSCLC, respectively.^15^, ^22^ MET amplification has been detected after anti‐EGFR antibody treatment of KRAS‐wild‐type colorectal cancer.^23^ In addition, activation of the HGF‐MET pathway has been reported as a clinically relevant resistance mechanism in a variety of tumors, including trastuzumab resistance in HER2‐positive breast cancer, BRAF inhibitor resistance in melanoma, and bevacizumab resistance in glioblastoma.^24^, ^25^, ^26^ In line with this evidence, we have shown that HGF, which can be produced by tumor‐associated fibroblasts, induced entrectinib resistance in NTRK1 rearranged colon cancer and ROS1 rearranged NSCLC models.

Much attention has been paid to the drug‐tolerant persisters (DTPs) of targeted drugs. Typically, targeted therapy causes dramatic tumor shrinkage, but small lesions remain consistent with DTPs and enlarged later by additional resistance mechanisms. The mechanisms by which DTPs emerge are being studied intensively. We reported that AXL and IGF‐1R could be involved in the emergence of DTPs in EGFR‐mutated NSCLC during treatment with osimertinib, a third‐generation EGFR‐TKI.^27^, ^28^ The findings of the present study suggest that HGF, which can exist in the tumor microenvironment, may be involved in the emergence of DTPs against entrectinib treatment. Since tumors with NTRK or ROS1 rearrangement are very rare, we could not evaluate the expression of HGF in clinical specimens from patients before and after entrectinib treatment. Such experiments may demonstrate the clinical relevance of this resistance mechanism.

Capmatinib is a highly specific MET inhibitor that has shown efficacy in clinical trials of NSCLC with MET exon 14 skipping mutations or MET amplification, especially in treatment‐naïve patients with MET exon 14 skipping mutations.^21^ In this study, the combined use of capmatinib and entrectinib circumvented the resistance caused by HGF‐producing fibroblast MRC‐5 in a subcutaneous tumor model with KM12SM cells. Recently, several MET‐selective inhibitors have been used in clinical trials to circumvent targeted drug resistance associated with MET activation. For instance, tepotinib and savolitinib were combined with gefitinib in EGFR‐TKI‐refractory EGFR‐mutated NSCLC patients who acquired EGFR‐TKI resistance associated with MET amplification. These trials show the promising efficacy and feasibility of MET‐TKI combined with EGFR‐TKIs.^29^, ^30^ Though the combination of capmatinib and entrectinib was feasible in our mouse model, toxicity as well as efficacy should be carefully evaluated if this combination is evaluated in future clinical trials.

The detection of resistance mechanisms is a key issue for patient selection on treatment to circumvent resistance. Among the HGF/MET‐mediated resistance mechanisms, MET amplification and MET exon 14 skipping mutations are detectable by clinically available methods, including next‐generation sequencing. However, the detection of HGF overexpression and MET activation without MET gene alterations is challenging because of the heterogeneity of HGF‐producing fibroblasts and instability of MET phosphorylation in tumor specimens.^31^ HGF exists in various tissues as a biologically inactive single‐chain polypeptide (scHGF) and is converted to functionally active two‐chain HGF (tcHGF) in the cancer microenvironment. HiP‐8 is a macrocyclic peptide that binds only to active HGF (tcHGF).^21^ Recently, we generated radiolabeled HiP‐8 (^64^Cu‐labeled HiP‐8‐PEG11) as a tracer for PET imaging and demonstrated in a mouse xenograft model that radiolabeled Hip‐8 accumulated selectively into the HGF‐producing tumor rather than HGF‐non‐producing tumor. These results indicate the utility of radiolabeled HiP‐8 as a tracer for detecting HGF‐induced targeted drug resistance in patients. Clinical trials are warranted to evaluate the usefulness of radiolabeled HiP‐8 by PET imaging in the selection of patients whose tumors acquired HGF‐induced resistance.

In conclusion, we demonstrated that growth factors in microenvironments, such as HGF, may induce entrectinib resistance in tumors with NTRK1 or ROS1 rearrangement. Our data further suggest the necessity of optimal combination with inhibitors of resistance‐inducing growth factors to maximize the therapeutic efficacy of entrectinib.

## AUTHOR CONTRIBUTIONS

K.S. and S.Y. supervised the study. Y.T., S.T., and S.Y. conceived and designed the experiments. Y.T., S.A., and C.S. carried out the experiments. Y.T., K.F, A.N., S.T. and S.Y. carried out data analysis and interpretation of data. H.S. and K.M. contributed technical, or material support. Y.T. and S.Y. drafted the manuscript. All authors have read and approved the manuscript.

## FUNDING INFORMATION

This work was supported by JSPS KAKENHI Grant Number JP16H05308 (to S. Yano) and JP20K09168 (to K. Sugio), the Project for Cancer Research And Therapeutic Evolution (P‐CREATE) from the Japan Agency for Medical Research and development, AMED, Grant Number 16cm0106513h0001 (to S. Yano), and Extramural Collaborative Research Grant of Cancer Research Institute, Kanazawa University.

## CONFLICT OF INTEREST

Dr. Yano obtained commercial research grants and speaking honoraria from Chugai Pharmaceutical. All the other authors have declared no conflicts of interest.

## ETHICS APPROVAL

The study protocol was approved by the Ethics Committee on the Use of Laboratory Animals and the Advanced Science Research Center, Kanazawa University, Kanazawa, Japan (approval no. AP‐173867).

## Data Availability

The data of this study are available from the authors upon reasonable request.
